# Association of sarcopenia, sarcopenic obesity with incident dementia, cognitive functions, and brain structure: findings from the UK Biobank Study

**DOI:** 10.1016/j.jnha.2026.100879

**Published:** 2026-05-19

**Authors:** Xinyue Zhang, Wenyang Han, Yiqun Li, Pinni Yang, Yiming Jia, Lulu Sun, Ruirui Wang, Mengyao Shi, Xiaowei Zheng, Yonghong Zhang, Zhengbao Zhu

**Affiliations:** a Public Health Research Center and Department of Public Health and Preventive Medicine, Wuxi School of Medicine, Jiangnan University, Wuxi, Jiangsu, 214122, China; b Department of Epidemiology, School of Public Health, Jiangsu Key Laboratory of Preventive and Translational Medicine for Major Chronic Non-Communicable Diseases, MOE Key Laboratory of Geriatric Diseases and Immunology, Suzhou Medical College of Soochow University, Suzhou, 215123, Jiangsu, China

**Keywords:** Alzheimer’s disease, Dementia, Sarcopenia, Sarcopenic obesity

## Abstract

**Background:**

Several small-sample studies have suggested that sarcopenia and sarcopenic obesity are implicated in cognitive decline. We aimed to prospectively investigate the associations of possible sarcopenia, sarcopenia and sarcopenic obesity with incident dementia, cognitive functions, and brain structure based on the UK Biobank.

**Methods:**

A total of 420,916 participants without dementia and cardiovascular diseases at baseline were analyzed. Sarcopenia status was defined according to the European Working Group on Sarcopenia in Older People 2. Obesity was defined according to body mass index. Cox models were applied to evaluate the longitudinal associations.

**Results:**

During a median follow-up of 13.69 years, 4,019 incident all-cause dementia events (including 2,650 Alzheimer’s disease and 527 vascular dementia) were recorded. Comparted with individuals without sarcopenia, the multivariable-adjusted hazard ratios (95% confidence interval) of all-cause dementia were 1.44 (1.32−1.58), 1.37 (1.03−1.82) and 3.06 (1.89−4.93) for those with possible sarcopenia, sarcopenia and severe sarcopenia, respectively. Individuals with sarcopenic obesity (hazard ratios = 1.69, 1.41−2.03) had the highest risk of all-cause dementia than those with obesity only, sarcopenia only or neither. The significant associations remained in all dementia types (Alzheimer’s disease or vascular dementia). In addition, those with sarcopenic obesity was associated with unfavorable cognitive functions, and worse brain structure.

**Conclusions:**

Our findings suggested that possible sarcopenia, sarcopenia and sarcopenic obesity were associated with a higher risk of all-cause dementia and its subtypes.

## 1 Introduction

Dementia, characterized by a progressive and unrelenting deterioration of mental capacity, is a major public health concern with around 50 million cases globally in 2019 [1,2]. With the accelerated ageing of the population, the number of dementia patients is anticipated to increase to 152 million by 2050 [3]. Due to the scarcity of curative treatment and the limited therapeutic value, identifying the modifiable risk factors of dementia is of high priority to reduce the disease burden of dementia [4].

Sarcopenia, describing a progressive and generalized skeletal muscle disorder involving the accelerated loss of muscle mass and function, is a public health concern with the physiological aging process [5,6]. The prevalence of sarcopenia ranges from 10% to 27% according to the recent European Working Group on Sarcopenia in Older People 2 (EWGSOP2) consensus definition [7,8]. In addition, the new concept of sarcopenic obesity, a co-occurrence of low muscle and excess body fat, has gained increasing focus [9,10]. Much of the evidence indicated that both sarcopenia and sarcopenic obesity were associated with increased risks of falls, functional decline, frailty, mortality in general population [7,11].

Emerging evidence had suggested the relationship between sarcopenia, sarcopenic obesity and dementia risk. Data from the Rush Memory and Aging Project (MAP) of 1,175 nondemented older adults indicated severe sarcopenia (defined according to grip strength and lean muscle mass) was associated with cognitive decline [12]. Evidence from the China Health and Retirement Longitudinal Study confirmed that sarcopenia (according to the Asian Working Group for Sarcopenia 2019 definition) is significantly associated with lower cognitive function and higher dementia risk score [13]. Moreover, the Bunkyo Health Study have shown close association between sarcopenic obesity and mild cognitive impairment (MCI) [14]. Taken together, the synthesis of previous reported studies pointed out that sarcopenia or sarcopenic obesity may be a potential predictor of dementia risk. However, due to the inconsistent of sarcopenia criteria, small sample size, and the limitations of methodological quality, the subsistent evidence remain insufficient to draw a definitive conclusion.

Based on the UK Biobank with large sample size and long-term follow-up, we conducted a prospective study to comprehensively investigate the associations of possible sarcopenia, sarcopenia, sarcopenic obesity with incident dementia. Furthermore, we tended to explored the associations of the possible sarcopenia, sarcopenia and sarcopenic obesity with 5 cognitive functions and 6 brain structural measures in the prospective study based on the UK Biobank.

## 2 Methods

### 2.1 Study design and participants

The UK Biobank is a population-based cohort comprising more than half a million individuals 40–69 years of age. The recruitment of participants was conducted between 2006 and 2010, and the participants were invited to attend 1 of the 22 centers located throughout England, Scotland, and Wales for baseline assessments, including completing baseline questionnaires, providing biological samples and undergoing physical examinations. The details of UK Biobank ‘s methodology, and objectives have been reported elsewhere [15]. The UK Biobank was constructed under ethical approval obtained by the North West Multi-Centre Research Ethics Committee (REC reference: 11/NW/03820) and all participants provided written informed consent prior to participation.

Among the initial sample of 502,394 participants, those who met all the following criteria were included: (1) without dementia at baseline (defined as dementia diagnosed prior to the date of baseline assessment), (2) without cardiovascular diseases at baseline (defined as heart failure, atrial fibrillation, ischemic heart disease, and stroke cases diagnosed prior to the date of baseline assessment), (3) with available records of sarcopenia and body mass index (BMI), (4) with records of dementia in follow-up. Finally, a total of 420,916 individuals remained for the final analysis. Supplementary Fig. S1 presents the illustration of the study workflow, and the detail of information of excluded participants was shown in Supplementary Table S1.

### 2.2 Assessment of sarcopenia and obesity/overweight

Muscle mass index was derived from skeletal muscle mass (kg) divided by height (m) squared using the total body composition measured via bioimpedance (BIA, Tanita BC418MA, Tokyo, Japan) by trained nurses. The estimate skeletal muscle mass was calculated according to the Janssen equation [16]. We used the cut-points for low muscle mass as recommended in the EWGSOP2 definition of <7 kg/m^2^ in men and 5.5 kg/m^2^ in women [7]. Grip strength at baseline was measured from both hands with the participant in the seated position and their forearms on armrests by a trained research nurse using a Jamar handheld dynamometer, and the maximum of values were included in the analysis [17]. As recommended in the EWGSOP2, the cut-points for low grip strength were <27 kg in men and <16 kg in women. The UK Biobank does not contain an objective measure of gait speed as specified by the EWGSOP2 criteria. Thus, self-reported walking pace was used as a proxy of gait speed and categorized as slow, average or brisk. Previous studies had proved that self-reported walking pace is a good marker of walking speed [18]. As a surrogate marker of poor gait speed and low performance, we considered participants who self-reported being unable to walk or their walking pace as ‘slow’.

Sarcopenia status in current study was assessed using three physical capability markers according to the recommended diagnostic algorithm of EWGSOP2 [7]. The possible sarcopenia was defined as low grip strength only (other physical capability markers in the normal range); sarcopenia was defined as low grip strength plus low muscle mass; and severe sarcopenia was defined as the combination of sarcopenia and slow gait speed [7]. BMI was calculated as weight (kg)/squared height (m^2^) and classified into three categories under the World Health Organization’s criteria: underweight and normal weight (<25 kg/m^2^), overweight (25 to <30 kg/m^2^), and obese (≥30 kg/m^2^).

### 2.3 Assessment of incident dementia, cognition and brain structure assessment

The outcome of present study was all-cause dementia (ACD) and its two major subtypes Alzheimer’s disease (AD) and vascular dementia (VaD). All diagnoses were recorded and classified under the International Classification of Diseases (ICD)-10 coding systems (ACD: F00, F01, F02, F03, F05.1, G30, G31.1, and G31.8; AD: F00 and G30; and VaD: F01) (Supplementary Table S2). The reliability of identifying dementia diagnoses in the UK biobank has been well validated, and shows high positive predictive value estimates of 80%–87% when combining all data sources [19]. The follow-up time was calculated from the date of recruitment until the date of earliest dementia incident, loss to follow-up, death, or linkage update from a general practitioner or inpatient admission.

The outcome of cognition included 5 cognitive functions (i.e., prospective memory, reaction time, ﬂuid intelligence, numeric memory, and incorrect pairs matching), which capture the prospective memory, processing speed, verbal and numerical reasoning, attention/working memory, and visuospatial memory of the participants, respectively (Supplementary Table S3). The test of cognition was tested via a touchscreen interface in the UK Biobank assessment center at a single time-point of the imaging visit during 2014 to 2019(20). For brain structure, 6 brain structural measures (i.e. volume of gray matter, volume of white matter, total brain volume, volume of white matter hyperintensities, volume of hippocampus, and volume of gray matter in hippocampus) obtained from magnetic resonance imaging (MRI) at a single time-point since 2014 were also included as the outcomes in current study according to previous study [[20], [21], [22]].

### 2.4 Covariates

Baseline touchscreen questionnaire are used to derive information on several potential confounders: age, gender, ethnicity, education, Townsend Deprivation Index, current smoking, drinking status, physical activity and medication use at baseline (cholesterol-lowering medication, antihypertensive medication, and insulin). Educational attainment was self-reported and was classified into a higher (college/university degree or other professional qualification) and lower education. The Townsend deprivation index is a composite measure of deprivation based on unemployment, non-car ownership, non-home ownership, and household overcrowding. It is derived from the residential postcode, with a negative value representing high socioeconomic status [23]. Smoking status was self-reported as never, former, or current smoking. Alcohol intake was self-reported and expressed as never, less than once a month, and at least once a month. Metabolic equivalent of tasks minutes/week (MET minutes/week) was used to represent the total physical activity, which was computed by the sum of walking and moderate and vigorous activity reporting from the modified version of the International Physical Activity Questionnaire [24]. Two measurements of systolic and diastolic blood pressure were taken using the Omron HEM-7015IT digital blood pressure monitor or a manual sphygmomanometer, and the mean of the two measurements was used for analysis. Hypertension was defined according to the American Heart Association 2017 guidelines, in which systolic blood pressure ≥140 mm Hg and/or diastolic blood pressure ≥90 mm Hg were considered positive. Diabetes mellitus was ascertained by the self-reported doctor’s diagnosis or the prior use of insulin. Apolipoprotein E (*APOE*) ε4 status was determined by the number of carriers through the genetic database.

### 2.5 Statistical analysis

Participants’ baseline characteristics are presented as percentages for categorical variables, as the means with standard deviation for normally distributed continuous variables and as medians with interquartile range for non-normally distributed variables. Participants were stratified into four groups according to the sarcopenia status (no sarcopenia, possible sarcopenia, sarcopenia and severe sarcopenia). Trends across four groups were tested by the generalized linear regression analysis for continuous variables and the Cochran-Armitage trend chi-square test for categorical variables, respectively. Pearson’s (for continuous variables) and Point-biserial (for dichotomized variables) correlation tests were used to assess the correlations of the four groups.

Kaplan-Meier cumulative incidence plots were generated to assess the associations of the sarcopenia status and sarcopenic obesity status (control; obesity/overweight only; sarcopenia only; sarcopenic obesity/sarcopenia and overweight) with incident dementia (ACD, AD and VaD) during follow-up, and the log-rank test was used for statistical assessment. We fitted three Cox-proportional hazard models to investigate the longitudinal associations of sarcopenia and sarcopenic obesity with the risk of incident dementia. Model 1 was unadjusted. Model 2 was adjusted for sociodemographic factors (age, sex, ethnicity, body-mass-index, and Townsend deprivation index) + lifestyle factors (smoking, alcohol, and MET minutes/week). Model 3 was further adjusted for disease risk factors (hypertension, diabetes, and *APOE* ε4 status) + medications (cholesterol-lowering medication, blood pressure medication, and insulin) and the covariates of Model 2. The proportional hazards assumptions were examined based on Schöenfeld residuals for all models. In sensitivity analyses, we first excluded participants with *APOE* ε4 status to test the associations without genetic influences. Then we excluded any outcome events that occurred within 5 years of follow-up to minimize the potential effects of reverse causation. Furthermore, we assessed the associations between sarcopenia components (low muscle mass alone, low physical performance alone and low muscle strength alone) and the risk of incident dementia. In subgroup analysis, the effects of sex and age on the associations of sarcopenia and sarcopenic obesity with risk of dementia were further considered. The multiplicative interaction terms were fitted to examine the potential effects of age, sex, and the *p* values were displayed in Supplementary Table S4.

We used multivariate logistic regression models (for prospective memory) and linear regression models (for reaction time, ﬂuid intelligence, numeric memory, incorrect pairs matching, volume of gray matter, volume of white matter, total brain volume, volume of white matter hyperintensities, volume of hippocampus, and volume of gray matter in hippocampus) to prospectively assess the associations of sarcopenia status and sarcopenic obesity status with cognitive functions and brain structure when appropriate. Both brain structural measure and cognitive function (except for prospective memory) were standardized for comparison. Two tailed *P* < 0.05 was considered statistical significance. The multiple imputation with the Markov chain Monte Carlo method was performed to assign any missing covariate data. All statistical analyses and figure preparation were conducted using SAS statistical software (version 9.4, Cary, NC) and GraphPad Prism version 8.00 (GraphPad Software, San Diego, CA, USA).

## 3 Results

### 3.1 Baseline characteristics

A total of 420,916 individuals from the UK Biobank were included in current study. Among the included individuals, 241,333 (57.34%) were women, 397,199 (94.37%) were White, and the mean (SD) age at baseline was 55.80 (8.07) years, and the prevalence of possible sarcopenia, sarcopenia and severe sarcopenia was 7.24%, 0.56% and 0.11%, respectively. Baseline characteristics of the participants stratified by sarcopenia status are provided in Supplementary Table S5.

### 3.2 Association between possible sarcopenia, sarcopenia, sarcopenic obesity with dementia incident, cognition and brain structure

**Fig. 1:**
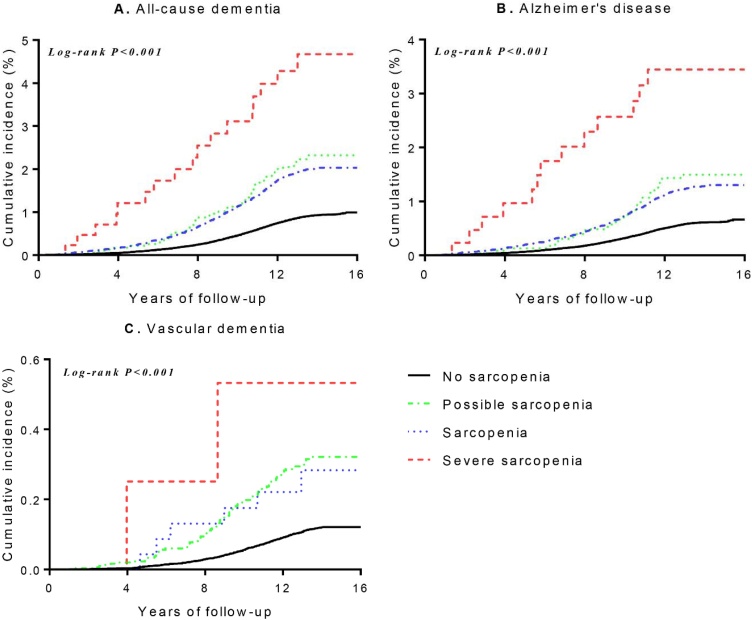
Cumulative Kaplan–Meier estimates of dementia according to the sarcopenia status. All-cause dementia; B. Alzheimer’s disease; C. Vascular dementia.

During a median follow-up of 13.69 years (12.96–14.40 years), 4,019 incident ACD events (including 2,650 AD and 527 VaD) were recorded. Kaplan-Meier curves indicated a graded increased risk of ACD, AD, and VaD in individuals with possible sarcopenia, sarcopenia and severe sarcopenia (all log-rank *P* < 0.001, Fig. 1). In unadjusted Cox regression analyses, possible sarcopenia, sarcopenia and severe sarcopenia were associated with increased risks of ACD, AD, and VaD, and these associations persisted after adjusting for sociodemographic and lifestyle factors. After further adjusted for disease risk factors, the hazard ratios (95% confidence intervals; HR; 95%CI) of ACD were 1.44 (1.32−1.58) for possible sarcopenia, 1.37 (1.03−1.82) for sarcopenia and 3.06 (1.89−4.93) for severe sarcopenia when comparted with those without sarcopenia (Table 1). Individuals with possible sarcopenia, sarcopenia and severe sarcopenia also had higher risk of AD and VaD (Table 1). When severe sarcopenia was divided into sarcopenia, individuals with possible sarcopenia and sarcopenia were also associated with increased risk of ACD, AD, and VaD (Supplementary Table S6). There were significant trends toward an increasing risk of ACD, AD, and VaD from the possible sarcopenia to severe sarcopenia (all *P* trend < 0.001).

**Table 1:** Prospective associations between the sarcopenia status and incident dementia.

	Sarcopenia status				*P* trend
	No sarcopenia	Possible sarcopenia	Sarcopenia	Severe sarcopenia	
Median of follow-up (year)	13.71	13.44	13.37	13.19	
All-cause dementia					
No. of cases, %	3,382 (0.87)	570 (1.87)	50 (2.11)	17 (3.83)	
Model 1, HR (95% CI)	1.00 (ref)	2.22 (2.03−2.43)	2.53 (1.91−3.34)	5.44 (3.38−8.76)	<0.001
Model 2, HR (95% CI)	1.00 (ref)	1.48 (1.35−1.62)	1.41 (1.06−1.87)	3.06 (1.89−4.94)	<0.001
Model 3, HR (95% CI)	1.00 (ref)	1.44 (1.32−1.58)	1.37 (1.03−1.82)	3.06 (1.89−4.93)	<0.001
Alzheimer’s disease					
No. of cases, %	2,239 (0.58)	365 (1.20)	33 (1.39)	13 (2.93)	
Model 1, HR (95% CI)	1.00 (ref)	2.14 (1.92−2.39)	2.51 (1.78−3.54)	6.25 (3.62−10.78)	<0.001
Model 2, HR (95% CI)	1.00 (ref)	1.53 (1.36−1.71)	1.46 (1.03−2.07)	3.56 (3.19−3.59)	<0.001
Model 3, HR (95% CI)	1.00 (ref)	1.49 (1.33−1.69)	1.43 (1.01−2.03)	3.55 (2.05−6.14)	<0.001
Vascular dementia					
No. of cases, %	429 (0.11)	90 (0.30)	6 (0.25)	2 (0.45)	
Model 1, HR (95% CI)	1.00 (ref)	2.69 (2.13−3.39)	2.92 (1.29−6.59)	5.03(1.25−20.31)	<0.001
Model 2, HR (95% CI)	1.00 (ref)	2.76 (2.20−3.46)	2.39 (1.07−5.34)	5.01 (1.25−20.09)	<0.001
Model 3, HR (95% CI)	1.00 (ref)	2.36 (1.87−2.98)	2.60 (1.15−5.88)	4.45 (1.10−17.97)	<0.001

Model 1: Unadjusted.

Model 2: Adjusted for sociodemographic factors (age, sex, ethnicity, body-mass-index, and Townsend deprivation index) + lifestyle factors (smoking, alcohol, and MET minutes/week).

Model 3: Adjusted for model 2 factors + disease risk factors (hypertension, diabetes, and APOE ε4 status) + medications (cholesterol-lowering medication, blood pressure medication, and insulin).

When comparing according to BMI and sarcopenia status, participants with sarcopenic obesity had the highest risk of ACD, AD, and VaD. Examination of the unadjusted effects of obesity, sarcopenia, and sarcopenic obesity on the risk of ACD, AD, and VaD compared with the normal group (without sarcopenia nor obesity) showed that there was a substantial elevated risk of ACD in participants with obesity only (HR = 1.17, 95% CI, 1.04−1.32), sarcopenia only (HR = 1.46, 95% CI, 1.32−1.61) and sarcopenic obesity (HR = 1.69, 95% CI, 1.41−2.03). These associations remained significant after full adjustment with all covariates (Table 2). Similarly, individuals with both sarcopenia and overweight were also associated with increased risks of ACD, AD, and VaD (Supplementary Table S7). As shown in Supplementary Table S8, individuals with low muscle mass alone, low muscle strength alone and low physical performance alone were significantly associated with increased risks of ACD, AD, and VaD, except for the association between low muscle mass alone and VaD.

**Table 2:** Prospective associations between the sarcopenia status, obesity status and incident dementia.

	Sarcopenia/obesity status*				*P* trend
	Control	Obesity only	Sarcopenia only	Sarcopenic obesity	
Median	13.45	13.36	13.01	12.98	
All-cause dementia					
No. of cases, %	2,623 (0.87)	759 (0.88)	471 (1.95)	166 (1.83)	
Model 1, HR (95% CI)	1.00 (ref)	1.02 (0.94−1.10)	2.32 (2.11−2.56)	2.19 (1.88−2.57)	<0.001
Model 2, HR (95% CI)	1.00 (ref)	1.19 (1.06−1.34)	1.49 (1.35−1.64)	1.78 (1.48−2.13)	<0.001
Model 3, HR (95% CI)	1.00 (ref)	1.17 (1.04−1.32)	1.46 (1.32−1.61)	1.69 (1.41−2.03)	<0.001
Alzheimer’s disease					
No. of cases, %	1753 (0.58)	486 (0.56)	315 (1.30)	96 (1.06)	
Model 1, HR (95% CI)	1.00 (ref)	0.98 (0.88−1.08)	2.32 (2.06−2.61)	1.89 (1.54−3.2)	<0.001
Model 2, HR (95% CI)	1.00 (ref)	1.21 (1.04−1.34)	1.51 (1.34−1.71)	1.66 (1.31−2.10)	<0.001
Model 3, HR (95% CI)	1.00 (ref)	1.19 (1.03−1.38)	1.49 (1.32−1.68)	1.58 (1.25−2.01)	<0.001
Vascular dementia					
No. of cases, %	316 (0.10)	113 (0.13)	63 (0.26)	35 (0.39)	
Model 1, HR (95% CI)	1.00 (ref)	1.26 (1.02−1.56)	2.57 (1.96−3.37)	3.83 (2.70−5.44)	<0.001
Model 2, HR (95% CI)	1.00 (ref)	1.04 (0.76−1.43)	2.58 (1.97−3.40)	3.16 (2.05−4.87)	<0.001
Model 3, HR (95% CI)	1.00 (ref)	1.01 (0.74−1.39)	2.30 (1.75−3.02)	2.64 (1.71−4.07)	<0.001

Model 1: Unadjusted.

Model 2: Adjusted for sociodemographic factors (age, sex, ethnicity, body-mass-index, and Townsend deprivation index) + lifestyle factors (smoking, alcohol, and MET minutes/week).

Model 3: Adjusted for model 2 factors + disease risk factors (hypertension, diabetes, and APOE ε4 status) + medications (cholesterol-lowering medication, blood pressure medication, and insulin).

* Sarcopenia included those with possible sarcopenia, sarcopenia and severe sarcopenia; Obesity: BMI ≥30 kg/m^2^.

Individuals with severe sarcopenia had worse performance in prospective memory, reaction time and numeric memory (all *P* value < 0.005; Table 3). Whole those with sarcopenic obesity had worse performance in all cognitive assessments (all *P* value < 0.005; Table 3), and were significantly associated with volume of gray matter, total brain volume, volume of white matter hyperintensities, volume of hippocampus, and volume of gray matter in hippocampus (Table 4).

**Table 3:** Associations of sarcopenia status, obesity status with cognition.

	Cognitive functions (N = 55,383 to 98,290)*^,^&				
	Prospective memory (OR [95% CI])	Reaction time (Beta [95% CI])	Fluid intelligence (Beta [95% CI])	Numeric memory (Beta [95% CI])	Incorrect pairs matching (Beta [95% CI])
Sarcopenia status					
No sarcopenia	1.00 (Ref)	1.00 (Ref)	1.00 (Ref)	1.00 (Ref)	1.00 (Ref)
Possible sarcopenia	1.44 (1.04−1.99)	−0.343 (−0.832, 0.147)	0.191 (−0.294, 0.677)	0.154 (−0.096, 0.404)	0.005 (−0.242, 0.253)
Sarcopenia	1.49 (1.35−1.64)	−0.535 (−1.001, −0.062)	0.194 (−0.275, 0.664)	0.204 (−0.031, 0.439)	−0.022 (−0.256, 0.211)
Severe sarcopenia	1.82 (1.03−5.23)	−0.871 (−1.343, −0.400)	0.343 (−0.125, 0.811)	0.331 (0.097, 0.565)	−0.138 (−0.371, 0.094)
Sarcopenia/obesity status					
Control	1.00 (Ref)	1.00 (Ref)	1.00 (Ref)	1.00 (Ref)	1.00 (Ref)
Obesity only	1.10 (1.03−1.17)	0.053 (−0.028, 0.134)	−0.023 (−0.102, 0.056)	0.020 (−0.034, 0.075)	0.005 (−0.070, 0.034)
Sarcopenia only	1.48 (1.27−1.72)	−0.329 (−0.384, −0.275)	0.130 (0.081, 0.179)	0.140 (0.106, 0.174)	−0.121 (−0.154, 0.088)
Sarcopenic obesity	1.64 (1.44−1.86)	−0.338 (−0.389, −0.287)	0.157 (0.104, 0.210)	0.146 (0.109, 0.183)	−0.134 (−0.169, −0.098)

* All expressed as z score units (standardized mean diﬀ ;erence), except prospective memory which was expressed as an odds ratio (OR).

& All analyses were adjusted for sociodemographic factors sociodemographic factors (age, sex, ethnicity, body-mass-index, and Townsend deprivation index) + lifestyle factors (smoking, alcohol, and MET minutes/week) + disease risk factors (hypertension, diabetes, and APOE ε4 status) + medications (cholesterol-lowering medication, blood pressure medication, and insulin).

**Table 4:** Associations of baseline, and clustering of eGDR with brain structure.

	Brain structure (N = 37,612 to 98,290)*^,^&					
	Volume of gray matter (Beta [95% CI])	Volume of white matter (Beta [95% CI])	Total brain volume (Beta [95% CI])	Volume of white matter hyperintensities (Beta [95% CI])	Volume of hippocampus (Beta [95% CI])	Volume of gray matter in hippocampus (Beta [95% CI])
Sarcopenia status						
No sarcopenia	1.00 (Ref)	1.00 (Ref)	1.00 (Ref)	1.00 (Ref)	1.00 (Ref)	1.00 (Ref)
Possible sarcopenia	−0.290 (−0.931, 0.352)	0.246 (−0.422, 0.914)	−0.052 (−0.705, 0.601)	−0.089 (−0.743, 0.564)	−0.087 (−0.752, 0.579)	0.070 (−0.595, 0.734)
Sarcopenia	−0.173 (−0.798, 0.453)	0.386 (−0.267, 1.038)	0.1033 (−0.533, 0.740)	−0.117 (−0.753, 0.519)	−0.040 (−0.689, 0.608)	193 (−0.455, 0.841)
Severe sarcopenia	0.013 (−0.610, 0.637)	0.533 (−0.117, 1.183)	0.307 (−0.328, 0.942)	−0.291 (−0.926, 0.344)	0.257 (−0.399, 0.578)	0.510 (−0.137, 1.156)
Sarcopenia/obesity status						
Control	1.00 (Ref)	1.00 (Ref)	1.00 (Ref)	1.00 (Ref)	1.00 (Ref)	1.00 (Ref)
Obesity only	0.023 (−0.069, 0.116)	0.008 (−0.088, 0.105)	0.020 (−0.074, 0.114)	−0.015 (−0.111, 0.080)	0.013 (−0.083, 0.108)	−0.015 (−0.111, 0.080)
Sarcopenia only	0.144 (0.086, 0.202)	0.168 (0.107, 0.229)	0.188 (0.129, 0.247)	−0.176 (−0.236, −0.116)	0.250 (0.185, 0.315)	0.241 (0.176, 0.306)
Sarcopenic obesity	0.299 (0.237, 0.362)	0.160 (0.094, 0.225)	0.284 (0.221, 0.348)	−0.194 (−0.259, −0.129)	0.342 (0.281, 0.402)	0.374 (0.314, 0.435)

* All expressed as z score units (standardized mean diﬀ ;erence).

& All analyses were adjusted for sociodemographic factors (age, sex, ethnicity, body-mass-index, and Townsend deprivation index) + lifestyle factors (smoking, alcohol, and MET minutes/week) + disease risk factors (hypertension, diabetes, and APOE ε4 status) + medications (cholesterol-lowering medication, blood pressure medication, and insulin).

### 3.3 Sensitivity analyses

When the analytic samples were limited to the follow-up period, the significant associations between possible sarcopenia, sarcopenia, sarcopenic obesity and the risk of ACD, AD, and VaD sustained. Individuals with possible sarcopenia, sarcopenia, sarcopenic obesity were associated with a 37%, 50% and 62% increased risk of ACD, respectively (Supplementary Table S9). Similarly, when the analytic samples were limited to negative *APOE* ε4 carrier status, possible sarcopenia, sarcopenia, sarcopenic obesity were associated increased risk of ACD, AD, and VaD (Supplementary Table S9).

### 3.4 Age and sex-specific analyses

In age-specific models, the least statistical consistency of the association between possible sarcopenia, sarcopenia, sarcopenic obesity and study outcomes were found among the age ≤55 subgroup (Supplementary Table S10). Among the age 56–65 and age >65 groups, individuals with sarcopenic obesity was associated with increased risk of ACD, AD, and VaD. Among the sex-specific models, only few distinctions were observed between genders. Possible sarcopenia, sarcopenia and sarcopenic obesity were associated with higher risks of all dementia types in both male and females, except for the association between sarcopenia and VaD in male (Supplementary Table S11).

## 4 Discussion

Based on the current large, prospective, population-based cohort of middle-aged individuals, our findings have demonstrated that possible sarcopenia, sarcopenia, sarcopenic obesity, and sarcopenia’ components (low muscle strength, low muscle mass and low physical performance) were associated with a higher risk of ACD, AD, and VaD, and the associations were statistically significant after adjustment for the well-established ACD risk factors. Furthermore, those with sarcopenic obesity were significantly associated with cognitive functions, and brain structure. Our study provided the valid evidence of the longitudinal relationship between sarcopenia/BMI status and risk of dementia.

In 2017, sarcopenia was recognized by the International Classification of Diseases (ICD-10-MC) as a progressive muscle disease. Actually, the diagnostic criteria for sarcopenia have long been unclear and most clinicians are not very clear about the diagnosis and treatment of the sarcopenia. Recently, the new concept of possible sarcopenia had been proposed [7,25], which may contribute to higher awareness of sarcopenia prevention and interventions in diverse health care settings. We found that the prevalence of possible sarcopenia, sarcopenia, severe sarcopenia and sarcopenic obesity was 7.24%, 0.56%, 0.11% and 2.16%, respectively, among UK Biobank participants aged 40–70 years. The data from UK Biobank also support the need to evaluate sarcopenia, especially possible sarcopenia and sarcopenic obesity in the general population. Considering the high prevalence of possible sarcopenia, sarcopenia and sarcopenic obesity, the insufficient diagnosis and treatment in clinical, as well as the poor prognosis of sarcopenia, our study provides evidences for future study in prevention and control of sarcopenia and sarcopenic obesity.

Several studies had suggested that the presence of sarcopenia was correlated with higher risks of AD, MCI, and cognitive decline [[12], [13], [14]]. Data from the China Health and Retirement Longitudinal Study of 1,978 participants (aged 65 years and older) demonstrated that sarcopenia (according to the AWGS 2019 definition) was significantly associated with lower cognitive function (standardized, β = −0.15; 95% CI: −0.26, −0.04) and a higher dementia risk score (standardized, β = 0.42; 95% CI: 0.29, 0.55) [13]. In a systematic meta-analysis of 15 studies, sarcopenia is associated with an increased risk of cognitive impairment independent of the study population, sarcopenia definition, and cognitive impairment definition [26]. Beeri et al. found more severe sarcopenia was associated with a higher risk of incident AD (HR = 1.50; 95% CI 1.20–1.86), MCI (HR = 1.21; 95%CI 1.01–1.45), and a faster rate of cognitive decline (β = 0.013) in a cohort study of 1,175 nondemented older adults with average follow-up of 5.6 years [12]. Although the diagnosis criteria for sarcopenia differ markedly with previous studies, we found that both sarcopenia and severe sarcopenia were significantly associated with ACD, AD, and VaD. Furthermore, we also found that individuals with possible sarcopenia had a 44%, 49% and 136% higher risk of ACD, AD, and VaD, respectively. To our knowledge, this is the first large longitudinal study to systematically assess the association between sarcopenia status and dementia risk. However, it is worth noting that the prevalence of severe sarcopenia was low (0.11%) in this middle-aged cohort, the estimated risk may be wider confidence intervals due to small subgroup sizes. Further research with larger sample sizes is needed to confirm our findings.

Although the universally recognized diagnostic criteria for sarcopenic obesity was not agreed [27,28], the sarcopenic obesity is appropriately characterized as a confluence of two epidemics-an ageing population and rising obesity rates [29]. Several studies had also reported that sarcopenic obesity is associated with a higher risk of cognitive impairment than either alone [14,[30], [31], [32]], despite of the different diagnostic criteria of sarcopenia and obesity. As part of current study, we also found that participants with sarcopenic obesity were significantly correlated with increased risks of ACD, AD, and VaD. In addition, when overweight measured by BMI ≥ 25 kg/m^2^, the combination of overweight and sarcopenia were still at higher risk of dementia risk. Our findings supported the validity of the association between sarcopenic obesity and dementia risk. Furthermore, our findings extended those evidence with finding that sarcopenic obesity was significantly with brain structural (6 brain structural measures). The present study provides a more valid appraisal of the relationship between sarcopenic obesity and cognition and brain structure.

Whether the EWGSOP2 or AWGS 2019 definition, sarcopenia status was assessed according to the three components: muscle strength, skeletal muscle mass (ASM), and physical performance [7,13]. A few previous studies have separately demonstrated the associations between three components and dementia risk. A cohort study had demonstrated that grip strength but not muscle mass was associated with the rate of cognitive decline [12]. Furthermore, the study found better grip strength was associated with a reduced risk of incident AD, but muscle mass was not associated with incident AD [12]. A study from the UK Biobank of 340,212 participants suggested that a 5 kg increment of absolute grip strength was associated with lower risks of ACD, AD, and VaD, while a slow walking pace (<3 miles per hour) was associated with increased risk of dementia risk [33]. In consistent with previous studies, we found component of sarcopenia (low muscle mass, low muscle strength or low physical performance) was associated with the incidence of ACD, AD, and VaD, except for the association between low muscle mass alone and VaD. Our findings supported the validity of current major criteria of sarcopenia by the EWGSOP2 criteria [7]. Previous studies and our results have proved that whether defined as a continuous or classified variable, muscle strength, ASM, and physical performance was a perfect predictor of sarcopenia, and the assessment of sarcopenia in community-based health check-ups and routine clinical practice might facilitate identification of those at greatest risk of potential incident dementia risk.

In stratified by gender, the associations between possible sarcopenia, sarcopenia, sarcopenic obesity and the risk of incident dementia remained significant in both male and female subgroups. These findings align with a previous report that showed significant association between grip strength (both absolute and relative terms), walking pace and dementia risk in both gender [33]. However, several studies showed that sarcopenia and sarcopenic obesity were significantly associated with MCI and dementia in females than males [14,34]. The inconsistency between studies may be due to the different sample sizes. Aging adults show changes in body composition and habitus with loss of lean muscle mass [35]. Both our study and other study from UK Biobank found the least statistical consistency about the exposure and dementia risk in the age ≤55-year subgroup. It is possible that the results were affected by the low prevalence of sarcopenia and low incidence of dementia in younger participants. Future researches should focus on the elderly individuals.

The potential mechanisms underlying the association between sarcopenia, sarcopenic obesity and dementia are multifaceted. First, sarcopenia reduces insulin sensitivity, leading to insulin resistance, and further interferes with the reninangiotensin-aldosterone system, which may induce hypertension with myocardial fibrosis [[36], [37], [38]]. Second, sarcopenia is a hyperinflammatory state and sarcopenic obesity is associated with muscle mitochondrial dysfunction, which may generate reactive oxygen species through oxidative stress, thereby damaging the vascular endothelium and cardiac myocyte [39]. Third, sarcopenia may damage the autonomic nerves, which in turn causes sympathetic excitation and greatly affects the heart and blood vessel [40]. Future studies are needed to explore exact mechanisms.

The current study was conducted based on UK Biobank, which was a large prospective cohort of middle-aged and older general population, with an unprecedented amount of biological and medical data. In addition, exposure, outcome, and covariates were measured according to standardized protocols and rigorous quality control procedures, which may contribute to valid evaluation of association between sarcopenia and dementia. However, several limitations need to be mentioned. First, the gait speed was evaluated by self-reported walking pace, and muscle mass was measured using BIA instead of the gold standard of dual-energy X-ray absorptiometry. However, previous studies have proved that self-reported walking pace was a simple and cheap marker of physical capability, and has a strong predictive ability for chronic diseases and mortality [41,42]. Similarly, muscle mass estimated by BIA has been proved to have good agreement with DXA (r = 0.868) [43]. Second, owing to low prevalence of sarcopenia, especially severe sarcopenia, therefore, we combined possible sarcopenia, sarcopenia and severe sarcopenia in sarcopenic obesity, and we were unable to study sarcopenia or severe sarcopenia as a separate category. Third, considering the observational design of this study, some of the associations identified might be affected residual confounding effects even though several major confounding factors were adjusted in our analyses. Finally, the incidents of dementia were determined by the ICD rather than the conventional neuropsychiatric screening. Although identification of dementia diagnoses via registries had been proved to be of high accuracy [19], the potential underdiagnosis particularly of early-stage or mixed dementias can’t be avoided.

In conclusion, our study based on UK Biobank demonstrated that possible sarcopenia, sarcopenia, severe sarcopenia and sarcopenic obesity was significantly associated with incident dementia, unfavorable cognitive functions, and worse brain structure, even after adjustment for a large range of potential confounders. Further studies are warranted to explore more effective interventions for sarcopenia.

## Key summary points

**Aim**

The aim was to investigate the associations of possible sarcopenia, sarcopenia, sarcopenic obesity with incident dementia, and to explored the associations of the possible sarcopenia, sarcopenia and sarcopenic obesity with 5 cognitive functions and 6 brain structural measures.

**Findings**

Based on UK Biobank, we demonstrated that possible sarcopenia, sarcopenia, severe sarcopenia and sarcopenic obesity was significantly associated with incident dementia, unfavorable cognitive functions, and worse brain structure, even after adjustment for a large range of potential confounders.

**Message**

Our findings provide more valid evidence for screening and preventing sarcopenia and obesity, which may be beneficial in reducing the incidence and disease burden of dementia.

## Author contributions

Conceptualization: Xiaowei Zheng and Zhengbao Zhu; Methodology: Xianyue Zhang, Yiqun, Li and Wenyang Han; Formal analysis and investigation Yiqun, Li and Wenyang Han; Writing—original draft preparation: Xianyue Zhang, Xiaowei Zheng; Writing-review and editing: Pinni Yang, Yiming Jia, Lulu Sun and Ruirui Wang; Resources: Mengyao Shi and Zhengbao Zhu; Supervision: Mengyao Shi and Zhengbao Zhu; All authors contributed to subsequent revisions and approved the final version. All authors read and approved the final manuscript.

## Consent for publication

Not applicable.

## Ethics approval and consent to participate

UK Biobank was constructed under ethical approval obtained by the North West Multi-Centre Research Ethics Committee (REC reference: 11/NW/03820) and all participants provided written informed consent prior to participation. The current analyses were carried out under Application Number 91185.

## Declaration of Generative AI and AI-assisted technologies in the writing process

During the preparation of this work, the authors did not use AI tools.

## Funding

This research did not receive any specific grant from funding agencies in the public, commercial, or not-for-profit sectors.

## Availability of data and materials

The dataset supporting the conclusions of this article is available in the public UK Biobank Resource (www.ukbiobank.ac.uk/).

## Declaration of competing interest

The authors declare that they have no competing interests.
