# Supplementary material for: Association of sarcopenia, sarcopenic obesity with incident dementia, cognitive functions, and brain structure: findings from the UK Biobank Study
Source: J Nutr Health Aging. 2026 May 19;30(7):100879. doi: 10.1016/j.jnha.2026.100879 (PMC13213763; doi:10.1016/j.jnha.2026.100879)
Supplement: Supplementary file 1 [file mmc1.docx]

**Supplementary Table 1.** Information of excluded participants

| **Variates** | **No. of sample met with exclusion criteria** |
| --- | --- |
| Missing dementia data | 62,392 |
| Baseline dementia | 226 |
| Baseline cardiovascular diseases | 33,261 |
| BMI | 3,107 |
| Sarcopenia | 81,478 |
| ^*^ Table represents the sample met with the exclusion criteria based on 502,394 participants. Note that some numbers are overlapped. | |

**Supplementary Table 2.** The Definition of Diseases.

| **Diagnosis** | **Code Type** | **Codes** |
| --- | --- | --- |
| All-cause dementia | ICD-10 | A81.0, F00, F00.0, F00.1, F00.2, F00.9, F01, F01.0, F01.1, F01.2, F01.3, F01.8, F01.9, F02, F02.0, F02.1, F02.2, F02.3, F02.4, F02.8, F03, F05.1, F10.6, G30, G30.0, G30.1, G30.8, G30.9, G31.0, G31.1, G31.8, I67.3 |
| Alzheimer’s disease | ICD-10 | F00, F00.0, F00.1, F00.2, F00.9, G30, G30.0, G30.1, G30.8, G30.9 Read V2 |
| Vascular dementia | ICD-10 | F01, F01.0, F01.1, F01.2, F01.3, F01.8, F01.9, I67.3 |

**Supplementary Table 3.** Description of cognitive tests

| **Cognitive test** | **Questions/measures** | **Domains** | **Ranges** | **Definition of better performance** |
| --- | --- | --- | --- | --- |
| Prospective memory | “At the end of the games we will show you four colored shapes and ask you to touch the Blue Square. However, to test your memory, we want you to actually touch the Orange Circle instead." | Prospective memory | 0 versus 1 | 1=correct at first attempt |
| Reaction time | Mean time to correctly identify matches of two cards (unit: millisecond) | Processing speed | 150-1809 | Faster reaction (reverse coded) |
| Fluid intelligence | 2 min to complete as many questions as possible; number of correctly answered items out of 13 questions | Verbal and numerical reasoning | 0-13 | Higher fluid intelligence score |
| Numeric memory | Memorize 2-digit number; maximum digits remembered correctly | Attention/working memory | 2-12 | More digits remembered correctly |
| Pairs matching | Match 3 pairs of 6 cards in 5 seconds in the first round and match 6 pairs of 12 cards in 5 seconds in the second round; number incorrect matches/errors | Visuospatial memory | 0-48 | More correct match (reverse coded) |

**Supplementary Table 4.** *P* values on interaction between exposures and stratification variables with dementia incidents.

|  | All-cause dementia^*^ | | Alzheimer’s disease^*^ | | | Vascular dementia^*^ | |
| --- | --- | --- | --- | --- | --- | --- | --- |
|  | Ag | Sex | Age | | Sex | Age | Sex |
| **Sarcopenia status** |  |  |  | |  |  |  |
| No sarcopenia | Reference | Reference | | Reference | Reference | Reference | Reference |
| Possible sarcopenia | <0.001 | <0.001 | | <0.001 | <0.001 | <0.001 | <0.001 |
| Sarcopenia | <0.001 | <0.001 | | <0.001 | <0.001 | <0.001 | <0.001 |
| **Sarcopenia^#^/ obesity status** | |  | |  |  |  |  |
| Control | Reference | Reference | | Reference | Reference | Reference | Reference |
| Obesity only | <0.001 | <0.001 | | <0.001 | <0.001 | <0.001 | <0.001 |
| Sarcopenia only | <0.001 | <0.001 | | <0.001 | <0.001 | <0.001 | <0.001 |
| Sarcopenic obesity | <0.001 | <0.001 | | <0.001 | <0.001 | <0.001 | <0.001 |

^*^Adjusted for sociodemographic factors (age, sex, ethnicity, body-mass-index, and Townsend deprivation index) + lifestyle factors (smoking, alcohol, and MET minutes/week) + disease risk factors (hypertension, diabetes, and APOE ε4 status) + medications (cholesterol-lowering medication, blood pressure medication, and insulin).

^#^Sarcopenia included those with possible sarcopenia, sarcopenia and severe sarcopenia.

**Supplementary Table 5.** Characteristics of the study participants according to sarcopenia status.

| Characteristics | Sarcopenia status | | | | *P* trend |
| --- | --- | --- | --- | --- | --- |
|  | No sarcopenia | Possible sarcopenia | Sarcopenia | Severe sarcopenia |  |
| No. of subjects | 387,624 | 30,482 | 2,366 | 444 |  |
| Age (mean, SD) | 55.52(8.07) | 59.01(7.34) | 60.40(6.78) | 59.19(7.43) | <0.001 |
| Male (%) | 169,063(43.62) | 9,812(32.19) | 569(24.05) | 139(31.31) | <0.001 |
| White ethnicity (%) | 367,059(94.69) | 27,646(90.70) | 2,106(89.01) | 388(87.39) | <0.001 |
| Higher education (%) | 153,035(39.48) | 9,182(30.12) | 798(33.73) | 105(23.65) | <0.001 |
| BMI, kg/m^2^ (mean SD) | 27.15(4.64) | 28.29(4.94) | 20.95(1.91) | 21.09(2.26) | <0.001 |
| Smoking (%) |  |  |  |  | <0.001 |
| Never | 218,720(56.43) | 17,439(57.21) | 1,455(61.50) | 191(43.02) |  |
| Previous smoker | 130,269(33.61) | 10,156(33.32) | 622(26.29) | 113(25.45) |  |
| Current smoker | 38,635(9.97) | 2,887(9.47) | 289(12.21) | 140(31.53) |  |
| Alcohol intake (%) |  |  |  |  | <0.001 |
| Never | 14,772(3.81) | 2,492(8.18) | 201(8.50) | 58(13.06) |  |
| Less than a month | 12,002(3.10) | 1,744(5.72) | 140(5.92) | 52(11.71) |  |
| Least once a month | 360,850(93.09) | 26,246(86.10) | 2,025(85.59) | 334(75.34) |  |
| Blood pressure |  |  |  |  |  |
| SBP, mm Hg (mean, SD) | 137.13(18.41) | 137.71(18.58) | 132.92(19.26) | 133.45(20.54) | 0.033 |
| DBP, mm Hg (mean, SD) | 82.32(10.05) | 81.55(9.92) | 77.10(9.77) | 77.97(10.42) | <0.001 |
| Diabetes mellitus (%) | 14,327(3.70) | 2,369(7.77) | 64(2.70) | 15(3.38) | <0.001 |
| Hypertension (%) | 96,251(24.83) | 10,175(33.38) | 446(18.85) | 122(27.48) | <0.001 |
| Medications (%) |  |  |  |  | <0.001 |
| Cholesterol-lowering medication | 35,767(9.23) | 3,946(19.51) | 254(10.74) | 60(13.51) |  |
| Blood pressure medication | 47,024(12.13) | 5,946(19.51) | 254(10.74) | 60(13.51) |  |
| Insulin | 5,792(1.49) | 194(0.64) | 17(0.72) | - |  |
| Townsend Deprivation Index (mean, SD) | -1.44(3.01) | -0.76(3.28) | -1.12(3.08) | 0.16(3.77) | <0.001 |
| MET minutes/week (mean, SD) | 61.12(55.74) | 59.77(46.36) | 60.69(47.66) | 60.40(25.03) | <0.001 |
| APOE ε4 (%) |  |  |  |  |  |
| 0 allele | 295,732(76.29) | 21,704(71.20) | 1,683(71.13) | 314(70.72) | <0.001 |
| 1 allele | 84,259(21.74) | 8,022(26.32) | 623(26.33) | 118(26.58) |  |
| 2 allele | 6,733(1.97) | 756(2.48) | 60(2.54) | 12(2.70) |  |

Data are presented as mean and SD for continuous variables and as frequency and % for categorical variables.

BMI: body mass index; SBP: systolic blood pressure; DBP: diastolic blood pressure; MET, Metabolic Equivalent of Tasks. APOE ε4, Apolipoprotein E4.

**Supplementary Table 6.** Prospective associations between the sarcopenia status and incident dementia.

|  | Sarcopenia status^*^ | | | *P* trend |
| --- | --- | --- | --- | --- |
|  | No sarcopenia | Possible sarcopenia | Sarcopenia |  |
| Median | 13.43 | 13.03 | 12.71 |  |
| **All-cause dementia** |  |  |  |  |
| No. of cases, % | 3,382(0.87%) | 570(1.87) | 67(2.38) |  |
| Model 1, HR (95% CI) | 1.00(ref) | 2.22(2.03-2.43) | 2.92(2.30-3.72) | <0.001 |
| Model 2, HR (95% CI) | 1.00(ref) | 1.48(1.35-1.62) | 1.64(1.28-2.09) | <0.001 |
| Model 3, HR (95% CI) | 1.00(ref) | 1.44(1.32-1.58) | 1.60(1.25-2.04) | <0.001 |
| **Alzheimer’s disease** |  |  |  |  |
| No. of cases, % | 2,239(0.58) | 365(1.20) | 46(1.64) |  |
| Model 1, HR (95% CI) | 1.00(ref) | 2.14(1.92-2.39) | 3.02(2.26-4.04) | <0.001 |
| Model 2, HR (95% CI) | 1.00(ref) | 1.46(1.31-1.64) | 1.66(1.23-2.23) | <0.001 |
| Model 3, HR (95% CI) | 1.00(ref) | 1.43(1.28-1.60) | 1.63(1.21-2.18) | <0.001 |
| **Vascular dementia** |  |  |  |  |
| No. of cases, % | 429(0.11) | 90(0.30) | 8(0.28) |  |
| Model 1, HR (95% CI) | 1.00(ref) | 2.76(2.20-3.46) | 2.75(1.36-5.53) | <0.001 |
| Model 2, HR (95% CI) | 1.00(ref) | 2.69(2.13-3.39) | 3.26(1.60-6.63) | <0.001 |
| Model 3, HR (95% CI) | 1.00(ref) | 2.36(1.87-2.98) | 2.90(1.42-5.91) | <0.001 |

^*^Those with severe sarcopenia were divided into sarcopenia.

Model 1: Unadjusted

Model 2: Adjusted for sociodemographic factors (age, sex, ethnicity, body-mass-index, and Townsend deprivation index) + lifestyle factors (smoking, alcohol, and MET minutes/week)

Model 3: Adjusted for model 2 factors + disease risk factors (hypertension, diabetes, and APOE ε4 status) + medications (cholesterol-lowering medication, blood pressure medication, and insulin)

**Supplementary Table 7.** Prospective associations between the sarcopenia status, overweight status and incident dementia.

|  | Sarcopenia/ overweight status^*^ | | | | *P* trend |
| --- | --- | --- | --- | --- | --- |
|  | Control | Overweight only | Sarcopenia | Sarcopenia/ overweight |  |
| Median | 13.71 | 13.70 | 13.40 | 13.44 |  |
| **All-cause dementia** |  |  |  |  |  |
| No. of cases, % | 1,184(0.87) | 2,198(0.87) | 220(2.02) | 417(1.86) |  |
| Model 1, HR (95% CI) | 1.00(ref) | 1.01(0.94-1.08) | 2.42(2.10-2.80) | 2.23(2.00-2.50) | <0.001 |
| Model 2, HR (95% CI) | 1.00(ref) | 0.83(0.75-1.07) | 1.48(1.28-1.71) | 1.23(1.08-1.41) | <0.001 |
| Model 3, HR (95% CI) | 1.00(ref) | 0.84(0.77-1.03) | 1.45(1.26-1.68) | 1.22(1.07-1.40) | <0.001 |
| **Alzheimer’s disease** |  |  |  |  |  |
| No. of cases, % | 793(0.58) | 1,446(0.57) | 1509(1.38) | 261(1.16) |  |
| Model 1, HR (95% CI) | 1.00(ref) | 0.99(0.91-1.08) | 2.46(2.06-2.93) | 2.08(1.81-2.39) | <0.001 |
| Model 2, HR (95% CI) | 1.00(ref) | 0.87(0.77-1.07) | 1.53(1.28-1.83) | 1.26(1.07-1.49) | <0.001 |
| Model 3, HR (95% CI) | 1.00(ref) | 1.19(1.03-1.38) | 1.49(1.32-1.68) | 1.58(1.25-2.01) | <0.001 |
| **Vascular dementia** |  |  |  |  |  |
| No. of cases, % | 133(0.10) | 296(0.12) | 25(0.23) | 73(0.33) |  |
| Model 1, HR (95% CI) | 1.00(ref) | 1.21(0.99-1.49) | 2.44(1.59-3.74) | 3.47(2.61-4.62) | <0.001 |
| Model 2, HR (95% CI) | 1.00(ref) | 0.88(0.68-1.15) | 2.37(1.54-3.63) | 2.53(1.82-3.56) | <0.001 |
| Model 3, HR (95% CI) | 1.00(ref) | 0.89(0.68-1.16) | 2.17(1.41-3.32) | 2.22(1.58-3.11) | <0.001 |

^*^ Sarcopenia included those with possible sarcopenia, sarcopenia and severe sarcopenia; Overweight: BMI≥ 25kg/m^2^

Model 1: Unadjusted

Model 2: Adjusted for sociodemographic factors (age, sex, ethnicity, body-mass-index, and Townsend deprivation index) + lifestyle factors (smoking, alcohol, and MET minutes/week)

Model 3: Adjusted for model 2 factors + disease risk factors (hypertension, diabetes, and APOE ε4 status) + medications (cholesterol-lowering medication, blood pressure medication, and insulin)

**Supplementary Table 8.** Prospective associations between the sarcopenia components and incident dementia.

| Exposure | All-cause dementia^*^ | | Alzheimer’s disease^*^ | Vascular dementia^*^ |
| --- | --- | --- | --- | --- |
| **All-cause dementia** |  |  | |  |
| Low muscle mass alone | 1.54(1.38-1.72) | 1.27(1.12-1.43) | | 1.25(1.1-1.42) |
| Low muscle strength alone | 2.28(2.09-2.48) | 1.49(1.37-1.63) | | 1.45(1.33-1.58) |
| Low physical performance alone | 2.31(2.30-2.75) | 2.03(1.85-2.23) | | 1.99(1.81-2.18) |
| **Alzheimer’s disease** |  |  | |  |
| Low muscle mass alone | 1.65(1.45) | 1.36(1.17-1.58) | | 1.35(1.16-1.56) |
| Low muscle strength alone | 2.21(1.99-2.46) | 1.55(1.39-1.72) | | 1.51(1.36-1.68) |
| Low physical performance alone | 2.24(1.99-2.51) | 1.90(1.68-2.14) | | 1.85(1.64-2.09) |
| **Vascular dementia** |  |  | |  |
| Low muscle mass alone | 1.37(0.99-1.89) | 1.42(0.99-2.02) | | 1.38(0.97-1.96) |
| Low muscle strength alone | 2.76(2.21-3.43) | 1.70(1.36-2.13) | | 1.62(1.30-2.03) |
| Low physical performance alone | 3.46(2.77-4.32) | 2.44(1.92-3.10) | | 2.30(1.81-2.91) |

Model 1: Unadjusted

Model 2: Adjusted for sociodemographic factors (age, sex, ethnicity, body-mass-index, and Townsend deprivation index) + lifestyle factors (smoking, alcohol, and MET minutes/week)

Model 3: Adjusted for model 2 factors + disease risk factors (hypertension, diabetes, and APOE ε4 status) + medications (cholesterol-lowering medication, blood pressure medication, and insulin)

**Supplementary Table 9.** Prospective associations of sarcopenia status, overweight status with dementia incidence in follow-up restricted models and APOE ε4 restricted models.

| Exposure | All-cause dementia^*^ | | Alzheimer’s disease^*^ | | Vascular dementia^*^ | |
| --- | --- | --- | --- | --- | --- | --- |
|  | Follow-up-restricted models | APOE ε4-restricted models | Follow-up-restricted models | APOE ε4-restricted models | Follow-up-restricted models | APOE ε4-restricted models |
| **Sarcopenia status** |  |  |  |  |  |  |
| No sarcopenia | 1.00(ref) | 1.00(ref) | 1.00(ref) | 1.00(ref) | 1.00(ref) | 1.00(ref) |
| Possible sarcopenia | 1.37  (1.24-1.5`) | 1.44  (1.32-1.58) | 1.33  (1.18-1.51) | 1.43  (1.28-1.60) | 2.30  (1.80-2.93) | 2.36  (1.87-2.98) |
| Sarcopenia | 1.50  (1.15-1.96) | 1.60  (1.25-2.04) | 1.54  (1.11-2.12) | 1.63  (1.21-2.19) | 2.41  (1.06-5.45) | 2.90  (1.42-5.91) |
| **Sarcopenia^#^/ obesity status** |  |  |  |  |  |  |
| Control | 1.00(ref) | 1.00(ref) | 1.00(ref) | 1.00(ref) | 1.00(ref) | 1.00(ref) |
| Obesity only | 1.15  (1.01-1.30) | 1.17  (1.04-1.32) | 1.13  (0.97-1.33) | 1.19  (1.03-1.38) | 0.96  (0.69-1.34) | 1.01  (0.74-1.39) |
| Sarcopenia only | 1.37  (1.23-1.52) | 1.46  (1.32-1.61) | 1.36  (1.19-1.56) | 1.49  (1.32-1.68) | 2.14  (1.59-2.87) | 2.30  (1.75-3.02) |
| Sarcopenic obesity | 1.62  (1.33-1.97) | 1.69  (1.41-2.03) | 1.48  (1.15-1.91) | 1.58  (1.25-2.01) | 2.56  (1.64-4.01) | 2.64  (1.71-4.07) |

^*^Adjusted for sociodemographic factors (age, sex, ethnicity, body-mass-index, and Townsend deprivation index) + lifestyle factors (smoking, alcohol, and MET minutes/week) + disease risk factors (hypertension, diabetes, and APOE ε4 status) + medications (cholesterol-lowering medication, blood pressure medication, and insulin).

^#^Sarcopenia included those with possible sarcopenia, sarcopenia and severe sarcopenia.

**Supplementary Table 10.** Prospective associations of with dementia incidence in age-specific models.

| Exposure | Age≤55 | | | Age 56-65 | | | Age>65 | | |
| --- | --- | --- | --- | --- | --- | --- | --- | --- | --- |
|  | All-cause dementia^*^ | Alzheimer’s disease^*^ | Vascular dementia^*^ | All-cause dementia^*^ | Alzheimer’s disease^*^ | Vascular dementia^*^ | All-cause dementia^*^ | Alzheimer’s disease^*^ | Vascular dementia^*^ |
| **Sarcopenia status** |  |  |  |  |  |  |  |  |  |
| No sarcopenia | 1.00(ref) | 1.00(ref) | 1.00(ref) | 1.00(ref) | 1.00(ref) | 1.00(ref) | 1.00(ref) | 1.00(ref) | 1.00(ref) |
| Possible sarcopenia | 1.84  (1.27-2.69) | 1.62  (1.04-2.54) | 3.25  (0.67-15.70) | 1.49  (1.28-1.73) | 1.59  (1.33-1.90) | 0.99  (0.14-7.18) | 1.33  (0.96-1.84) | 1.15  (0.82-1.61) | 1.46  (1.09-1.95) |
| Sarcopenia | 4.33  (1.75-3.41) | 4.31  (1.57-11.85) | 7.84  (1.59-38.62) | 1.30  (0.99-1.69) | 1.95  (1.20-3.18) | 2.15  (1.45-3.21) | 1.36  (1.21-1.53) | 0.96  (0.74-1.24) | 1.77  (0.78-4.05) |
| **Sarcopenia^#^/ obesity status** | |  |  |  |  |  |  |  |  |
| Control | 1.00(ref) | 1.00(ref) | 1.00(ref) | 1.00(ref) | 1.00(ref) | 1.00(ref) | 1.00(ref) | 1.00(ref) | 1.00(ref) |
| Obesity only | 1.66  (1.12-2.46) | 1.95  (1.25-3.04) | 2.27  (0.32-15.94) | 1.10  (0.92-1.33) | 1.11  (0.88-1.39) | 0.89  (0.52-1.54) | 1.17  (0.99-1.38) | 1.14  (0.92-1.42) | 1.13  (0.76-1.68) |
| Sarcopenia only | 2.30  (1.52-3.48) | 2.39  (1.51-3.80) | 8.68  (2.05-36.64) | 1.55  (1.32-1.83) | 1.73  (1.42-2.10) | 1.75  (1.06-2.91) | 1.32  (1.16-1.50) | 1.23  (1.05-1.46) | 1.41  (1.01-1.98) |
| Sarcopenic obesity | 2.47  (1.23-4.95) | 1.63  (0.62-4.30) | - | 1.57  (1.16-2.12) | 1.46  (1.00-2.14) | 2.48  (1.22-5.07) | 1.73  (1.35-2.21) | 1.68  (1.22-2.30) | 1.87  (1.08-3.23) |

^*^Adjusted for sociodemographic factors (age, sex, ethnicity, body-mass-index, and Townsend deprivation index) + lifestyle factors (smoking, alcohol, and MET minutes/week) + disease risk factors (hypertension, diabetes, and APOE ε4 status) + medications (cholesterol-lowering medication, blood pressure medication, and insulin).

^#^Sarcopenia included those with possible sarcopenia, sarcopenia and severe sarcopenia.

**Supplementary Table 11.** Prospective associations of physical activity and sleep scores with dementia incidence in sex-specific models.

| Exposure | Male | | | Female | | |
| --- | --- | --- | --- | --- | --- | --- |
|  | All-cause dementia^*^ | Alzheimer’s disease^*^ | Vascular dementia^*^ | All-cause dementia^*^ | Alzheimer’s disease^*^ | Vascular dementia^*^ |
| **Sarcopenia status** |  |  |  |  |  |  |
| No sarcopenia | 1.00(ref) | 1.00(ref) | 1.00(ref) | 1.00(ref) | 1.00(ref) | 1.00(ref) |
| Possible sarcopenia | 1.23  (1.09-1.39) | 1.40  (1.25-1.58) | 1.93  (1.53-2.44) | 1.80  (1.57-2.06) | 1.43  (1.28-1.60) | 2.36  (1.87-2.98) |
| Sarcopenia | 1.43  (1.05-1.95) | 1.49  (1.09-2.04) | 1.96  (0.92-4.18) | 1.94  (1.30-2.91) | 1.63  (1.21-2.19) | 2.90  (1.42-5.91) |
| **Sarcopenia^#^/obesity status** |  |  |  |  |  |  |
| Control | 1.00(ref) | 1.00(ref) | 1.00(ref) | 1.00(ref) | 1.00(ref) | 1.00(ref) |
| Obesity only | 1.08  (0.92-1.28) | 1.12  (0.96-1.32) | 1.02  (0.74-1.40) | 1.30  (1.09-1.54) | 1.19  (1.03-1.38) | 1.01  (0.74-1.39) |
| Sarcopenia only | 1.27  (1.12-1.45) | 1.42  (1.25-1.61) | 1.79  (1.35-2.37) | 1.77  (1.52-2.06) | 1.49  (1.32-1.68) | 2.30  (1.75-3.02) |
| Sarcopenic obesity | 1.30  (1.01-1.66) | 1.57  (1.23-2.01) | 2.30  (1.49-3.56) | 2.46  (1.87-3.23) | 1.58  (1.25-2.01) | 2.64  (1.71-4.07) |

^*^Adjusted for sociodemographic factors (age, sex, ethnicity, body-mass-index, and Townsend deprivation index) + lifestyle factors (smoking, alcohol, and MET minutes/week) + disease risk factors (hypertension, diabetes, and APOE ε4 status) + medications (cholesterol-lowering medication, blood pressure medication, and insulin).

^#^Sarcopenia included those with possible sarcopenia, sarcopenia and severe sarcopenia.


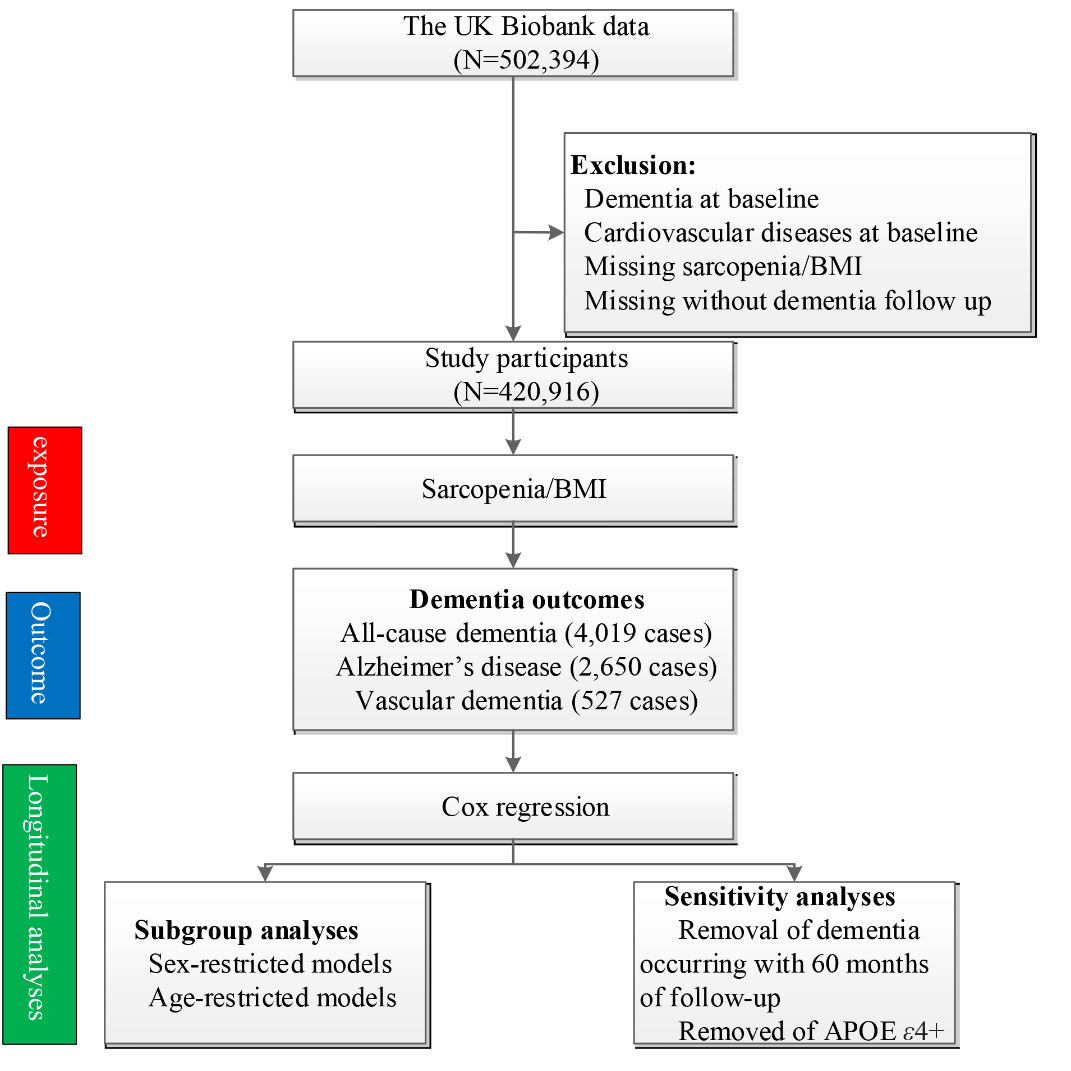


**Supplementary Figure 1.** Study workflow
